# Non-Coding RNAs in Endometrial Physiopathology

**DOI:** 10.3390/ijms19072120

**Published:** 2018-07-20

**Authors:** Alessandro La Ferlita, Rosalia Battaglia, Francesca Andronico, Salvatore Caruso, Antonio Cianci, Michele Purrello, Cinzia Di Pietro

**Affiliations:** 1 Department of Biomedical and Biotechnological Sciences, Biology and Genetics Section G. Sichel, University of Catania, 95123 Catania, Italy; alessandrolf90@hotmail.it (A.L.F.); rosaliabattaglia04@gmail.com (R.B.); francesca.andronico@gmail.com (F.A.); purrello@unict.it (M.P.); 2 Department of General Surgery and Medical Surgical Specialties, University of Catania, 95123 Catania, Italy; scaruso@unict.it (S.C.); acianci@unict.it (A.C.)

**Keywords:** miRNA, lncRNAs, endometrial cancer, endometriosis, chronic endometritis

## Abstract

The Human Genome Project led to the discovery that about 80% of our DNA is transcribed in RNA molecules. Only 2% of the human genome is translated into proteins, the rest mostly produces molecules called non-coding RNAs, which are a heterogeneous class of RNAs involved in different steps of gene regulation. They have been classified, according to their length, into small non-coding RNAs and long non-coding RNAs, or to their function, into housekeeping non-coding RNAs and regulatory non-coding RNAs. Their involvement has been widely demonstrated in all cellular processes, as well as their dysregulation in human pathologies. In this review, we discuss the function of non-coding RNAs in endometrial physiology, analysing their involvement in embryo implantation. Moreover, we explore their role in endometrial pathologies such as endometrial cancer, endometriosis and chronic endometritis.

## 1. Introduction

**Figure 1:**
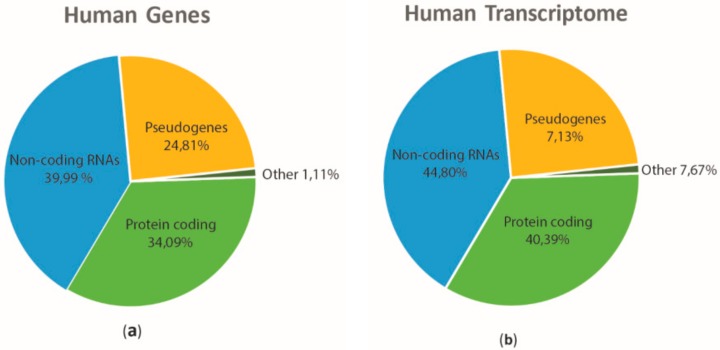
Overview of functional elements of the human genome and of the associated transcriptome. Pie chart showing the percentage of human genes (**a**) and transcripts (**b**) based on the current Release (Release 28, GRCh38.p12) of GENCODE. Classification is referred to Gene/Transcript Biotypes in GENCODE & Ensembl and to VEGA descriptions (available online: http://vega.archive.ensembl.org/info/about/gene_and_transcript_types.html). The non-coding transcriptome includes both small and long non-coding RNAs; pseudogenes incorporate processed, unprocessed, transcribed, translated, polymorphic and unitary sequences; other includes IG/TCR and their pseudogenes, together with non-stop decay and non-sense mediated decay.

At the beginning of the Human Genome Project (HGP), in the late 1990s, researchers hypothesized that our genome comprised about 100,000 protein-coding genes [1]. Over the years, this number has continuously decreased. In 2001, the International Human Genome Sequencing Consortium (IHGSC) published the initial sequence of the human genome and proposed that the number of protein-coding genes was about 30,000 [2]. The end of the sequencing of the human genome in 2004 revealed that the number of genes encoding for proteins were only 20–25,000 [3]. The latest version of human GENCODE (available online: https://www.gencodegenes.org/) established that the number of genes encoding for protein was 19,901 that is the 34.09% of human genes (Figure 1a). In spite of the low number of gene encoding proteins the ENCODE project, based on High Throughput Sequencing technologies and advances in bioinformatics, has provided a detailed landscape of transcription in human cells. (Figure 1b).

**Figure 2:**
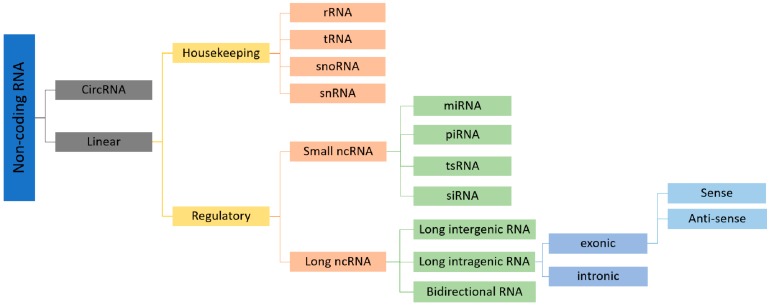
Schematic diagram illustrating the classification of ncRNAs according to their biological role and their length.

The non-coding RNAs (ncRNAs) could be classified, according on their size, into small ncRNAs (less than 200 nucleotides in length) and in long ncRNAs [(lncRNAs) longer than 200 nucleotides]. Alternatively, a further classification based on their function, split the ncRNAs into housekeeping and regulatory: the housekeeping ncRNAs, including ribosomal RNA (rRNA), transfer RNA (tRNA), small nuclear RNA (snRNA) and small nucleolar RNA (snoRNA), are expressed in all cell types and carry out essential functions in the cells, while the regulatory ncRNAs, including several classes of small and long molecules, assist in the regulation of gene expression, controlling different points of the central dogma (Figure 2).

The interest of scientific literature in regulatory ncRNAs is generated by the important roles that these molecules perform regulating cell proliferation, differentiation, migration, cell death and angiogenesis. Consequently, their altered expression is involved in different human pathologies. Another interesting characteristic for the potential implications for human health is that ncRNAs are present in all biological fluids associated with protein complexes or enclosed within extracellular vesicles (EVs) such as microvesicles or exosomes. Extracellular ncRNAs also show altered expression in different human pathologies, thus their role has been proposed as non-invasive biomarkers, prognostic factors and also therapeutic targets in cancer or in other complex diseases. Among the regulatory ncRNAs we found: microRNAs (miRNAs), small interfering RNAs (siRNAs), Piwi-associated RNAs (piRNAs), long non-coding RNAs (lncRNAs), circular RNAs (circRNAs) and the tRNA derived small RNAs (tsRNAs) (Figure 2). To date, the most studied molecules are miRNAs, lncRNAs and circRNAs. miRNAs are long 18–25 nucleotide (nt) single-stranded RNAs, evolutionarily conserved, which negatively modulate the expression of their target mRNAs. They bind to the 3′ Untranslated Region (3′ UTR) of specific mRNA targets, leading to translational repression, or mRNA cleavage [4,5,6] miRNAs are very important molecules in the regulation of gene expression at the post-transcriptional level, a single miRNA can control the expression of several mRNAs and a single mRNA may be targeted by more than one miRNA, thus creating a complex network of cooperative regulation [7]. lncRNAs are the most heterogeneous class of non-protein-coding RNAs with lengths ranging from 200 to 100,000 nt. They include transcripts that may be classified as: (a) intergenic lncRNAs; (b) intronic lncRNAs; (c) sense or antisense transcripts; (d) pseudogenes; and (e) retrotransposons [8]. lncRNAs regulate gene expression at different levels, including chromatin modification, alternative splicing, protein localization and activity and can protect 3′ UTR of mRNAs from miRNA binding, increasing their stability [8]. Several recent studies have also shown that lncRNAs are critically involved in a wide range of biological processes, such as cell cycle regulation, pluripotency, differentiation and cell death [9,10,11,12]. CircRNAs are a recently discovered class of circular single strand RNA molecules, covalently closed, which are resistant by the exonuclease [13]. Their function and the biological process in which they are involved remain mainly unknown but recent evidence suggests that circRNAs may play an important role in RNA–RNA interactions. In some cases, circRNAs exhibit multiple binding sites for the same miRNA and for this reason it has been suggested that they could represent potential molecular sponges for sequestering the most abundant miRNAs [14]. It means that they can act as a negative regulation of miRNAs by competing, using their binding sites, with the miRNA–mRNA target interaction. Their important role in gene regulation is demonstrated by some papers, which show that deregulation of circRNAs is associated in different types of cancer [15].

Scientific community recognized the important role played by regulatory ncRNAs in cell physiology and also the effects caused by their altered expression in all human diseases but many other studies will be necessary. In fact, sometimes, we are able to identify the differential expression of one or more ncRNAs in a particular disease and also to evaluate their prognostic value but we are not able to correlate accurately specific expression profiles to specific phenotype alteration, because of the complexity and the redundancy of the circuits regulating gene expression.

In this review, we will point up the ncRNA, particularly miRNA and lncRNAs, found expressed in endometrium in physiological conditions, focusing on their involvement in embryo implantation. Moreover, we will analyse the alteration of their expression in the pathogenesis of some endometrial diseases, as endometrial cancer, endometriosis and chronic endometritis, trying to understand how their altered expressions can influence cell proliferation, differentiation and apoptosis.

## 2. ncRNAs and Embryo Implantation

### 2.1. Embryo Implantation

The success of embryo implantation is related to blastocyst quality as well as to the endometrium receptivity. Both the embryo and the maternal tissues are able to reciprocally exchange signals; the proper secretion of signal molecules and their uptake allow the successful implantation [16,17]. 

Several mediators such as growth factors, cytokines, chemokines, lipids, matrix-degrading enzymes and integrins, whose expression is regulated by oestrogen (E) and progesterone (P), influence endometrial receptivity [18]. Many genes have been discovered that have important roles in embryo implantation and in 2011 a genomic tool was developed to assess the endometrial receptivity of woman during In Vitro Fertilization cycles (IVF). The Endometrial Receptivity Array (ERA) test, based on microarray technology, analyses the expression profile of 238 genes encoding proteins which are related with the implantation process, during the Window of Implantation (WOI). The ERA test has been shown to be more accurate than histological evaluation to assess endometrial receptivity. This has subsequently led to the new concept of personalized embryo transfer, by using endometrial biomarkers as a therapeutic strategy for patients with recurrent implantation failure [18,19,20,21,22] According to the rising interest in the role of ncRNAs in the regulation of gene expression, different studies have explored the involvement of miRNAs in implantation, focusing on the dialog between embryo and maternal tissues. Different miRNAs, associated with endometrial receptivity, have been identified in endometrial biopsies as well as in endometrial fluids (Table 1).

### 2.2. miRNAs in Endometrial Receptivity

Different studies, on animal models and humans, confirmed the important role played by miRNAs in endometrial physiology by regulating the changes in gene expression levels during the different phases of the endometrial cycle. In fact, many of them were found differentially expressed at each stage of the endometrial cycle.

MiR-30b and miR-30d were found to be significantly up-regulated and miR-494 down-regulated in the receptive endometrium (LH+7) compared with the pre-receptive endometrium (LH+2) from healthy fertile women. The bioinformatic prediction of the target genes of these miRNAs showed that they are involved in the cyclic remodelling of the endometrium, including endometrial maturation to the receptive state [23]. Mucin 1 (Muc1) is an integral transmembrane mucin glycoprotein expressed on the apical surface of the endometrium, acting as an inhibitor of embryo attachment. In mice, the expression of Muc1 decreased significantly during the WOI and could be due to a negative regulation mediated by miR-199a, let-7a and let-7b [24,25]. Insulin-like growth factor 1 receptor (IGF1R) is an important receptor, up-regulated in the endometrium during the receptive stage, which is closely related to embryo implantation. In fact, its increase on the surfaces of the endometrium might contribute to adhesive interaction with the embryo. It has been demonstrated that a high expression of miR-145 having as target the mRNA coding for IGF1R, inhibits embryo attachment. Mir-145 up-regulation has been shown in the endometrium of RIF patients [26].

Another very important characteristic for endometrial receptivity is the epithelial-mesenchymal transition (EMT). In EMT, the cells lose their polarity and display a remodelling of cell junctions in order to facilitate the interaction with embryo trophectoderm [27]. As an important suppressor of EMT, miR-429 exhibited a down-regulation during implantation in mice. Enhancement of miR-429 resulted in suppression of the migratory and invasive capacities of cells, probably through targeting protocadherin 8, leading to reduced implantation sites [28]. On the contrary, miR-126-3p was specifically up-regulated in implantation sites, promoting cell migratory and invasive capacity by regulating the expression of integrin α11 [29]. Moreover, we know that sex hormones induce changes in miRNA expression in the endometrium, for example, progesterone induced the expression of miR-125b in human endometrial epithelium cells. The increased expression of miR-125b inhibits cell movement and prevents implantation, by suppressing the expression of Matrix Metallopeptidase 26 (MMP26), a member of the matrix metalloproteinase family, which is involved in degradation of the extracellular matrix [30]. These studies demonstrated that, in addition to the cyclical changes in endometrium morphology and gene expression, there exists a very complex network of miRNAs regulating the expression of many genes encoding proteins related to endometrial receptivity and implantation.

### 2.3. Extracellular miRNAs in Endometrial Fluid

To assess endometrial receptivity, many researchers are focusing their studies on extracellular ncRNAs present in endometrial fluid. Endometrial fluid is a viscous fluid secreted by the endometrial glands into the uterine cavity and its role consists in nurturing the embryo and represents the important microenvironment in which both embryo and endometrium can interact with each other [31]. The embryo, induced by cytokines and different proteins secreted by the endometrium, modulates, in turn, the secretion of integrins β3, α4 and α1, interleukins, chemokines and leptine. Recently, ncRNAs have been added, as new players, in mediating the dialog between embryo and endometrium. It has been demonstrated that endometrial cells secrete exosomes in endometrial fluids and the secretion reaches a peak during WOI [32]. On the other hand, also embryos secrete extracellular vesicles in vitro that are able to be up taken by cultured endometrial cells [33]. During the last few years, the presence of miRNAs in endometrial fluid has been shown in humans and in animal models [27]. Comparing miRNA profiles between endometrial cells and exosomes present in endometrial fluid, 13 miRNAs out of 227 were exclusively present in exosomes/microvesicles and their mRNA targets were involved in several signalling pathways associated with implantation [34]. In 2015, in a very interesting paper, the authors analysed the RNA transcript present in endometrial fluid by microarray and found that a large number of miRNAs were present in the endometrial fluid. They found 27 miRNAs differentially expressed during the WOI with respect to the four different phases of the menstrual cycle and among them miR-30d was the most up-regulated miRNA [32]. The authors also demonstrated that miR-30d is an exosomal miRNA and it is internalized by trophoblastic cells of murine embryos. Moreover, embryos treated with miR-30d exhibited increased expression of ten genes, including those encoding adhesion molecules such as Integrin Subunit Beta 3 (ITGB3), Integrin Subunit Alpha 7 (ITGA7) and Cadherin 5 (CDH5) [32]. These studies demonstrated the molecular dialog, mediated by miRNAs, between embryo and endometrium, in order to promote embryo implantation.

## 3. ncRNAs and Endometrial Cancer

### 3.1. Endometrial Cancer

Endometrial cancer (EC) is the most common gynaecological tumour in developed countries, it is the fifth most common cancer and the 14th in terms of mortality [35]. It is becoming clear that EC, as well as many other tumours, includes different subtypes having specific genetic and molecular features. Based on histological characteristics, specific protein expression and grade, EC has been classified into two subtypes [36]. Type 1, also called endometrioid type (EEC), represents 70–80% of new sporadic cases. These cancers are typically well differentiated, associated with increasing oestrogen levels and the patients have a favourable prognosis. On the molecular level, more than 80% type I tumours are associated with a decreased or lacking expression of Phosphatase and tensin homolog (PTEN) and to an overexpressed Oestrogen Receptor (ER), which promote a deregulated cellular proliferation [37,38,39,40]. Furthermore, EEC are frequently associated with microsatellite instability (leading to DNA mismatch repair), genetic mutations and epigenetic abnormalities [41]. Type 2, known as non-endometrioid endometrial carcinoma (NEEC), is not related to circulating oestrogen levels and it is less common than type 1 (10–20% of ECs) [42]. Patients with NEEC, usually have a poor prognosis because the tumour is typically diagnosed when metastases are already present [36]. On the molecular level, NEEC tissue samples show a high expression of Tumour Protein p53 (TP53) and Cyclin Dependent Kinase inhibitor 2A (CDKN2A or P16), resulting in non-functional proteins that accumulate in the cell acting as a double negative inhibitor of the wild-type p53, leading to propagation of aberrant cells and to uncontrolled cell growth [43].

Many papers have investigated the role of ncRNA in EC in an attempt to find new molecular markers able to discriminate the different subtypes, predict prognosis and design new drugs for personalized targeted therapies [44,45,46,47]. In this review, we focused our attention on more important biological pathways and processes altered in EC and we discussed the role of ncRNAs in their regulation.

### 3.2. miRNAs in Endometrial Cancer

The phosphatidylinositol 3-kinase (PI3K) pathway is involved in the control of growth, survival, proliferation and apoptosis. Gain- or loss-of-function mutations of genes encoding proteins involved in the pathway lead to neoplastic transformations and it has been demonstrated that several components are dysregulated also in EC [48]. PTEN is a tumour suppressor that negatively regulates the PI3K-AKT signalling pathway. Loss of its function is implicated in the pathogenesis of a number of different tumours, including endometrial carcinoma [49]. PTEN mRNA is negatively regulated by miR-205 and it has been demonstrated that miR-205 up-regulation leads to increased B-cell lymphoma 2 (BCL2) levels and TP53 down-regulation, inhibiting tumour cell apoptosis and enhancing proliferation [50]. Moreover, by stimulating the AKT pathway and inhibiting glycogen synthase kinase 3β (GSK3B), miR-205 suppresses E-cadherin expression and promotes SNAIL expression [51]. Transfecting endometrial cancer cells with LNA-miR-205-inhibitor (Locked Nucleic Acid-inhibitor of miR-205), Torres et al. recently obtained decreased endometrial cancer cell proliferation in vitro and in vivo [52]. PTEN can also be controlled by miR-21, involved in enhanced malignant transformations and proliferation of type 1 EC cells [53]. The transcription factor Forkhead Box O1 (FOXO1), belonging to the Forkhead Box class O family, is a downstream target of the PI3K pathway and it is able to regulate different biological processes involved in endometrial phases such as menstruation, uterine cell regeneration, tissue remodelling and cell differentiation [54,55]. In EC type 1, FOXO1 is down-regulated and it has been shown that miR-9, miR-27, miR-96, miR-153 and miR-182, able to target FOXO1 mRNA, are over expressed in the same cells [56]. On the other hand, Forkhead Box C1 (FOXC1), another member of the forkhead box transcription factors, is an oncogene controlling tumour cell migration and metastasis and it is inhibited by miR-204 [57]. MiR-204 down-expression has been associated with solid tumour development, such as in the lung and gastric and endometrioid endometrial cancer [57,58,59]. The hyperactivation of the PI3K pathway leads to an inhibition of apoptosis by the repression of BCL2 associated agonist of cell death (BAD) and the enhancement of BCL2 activity [60]. On the other hand, apoptosis inhibition in EC can occur through BCL2 overexpression and BCL2 associated X (BAX) downregulation promoted by higher oestrogen levels. One of the most important regulator families involved in this mechanism seems to be the let-7 miRNA family, that is, let-7a, let-7b, let-7c, let7d, let-7e, let-7f and let-7g, whose expression grows in response to oestrogen exposure [61]. Nevertheless, high levels of let-7 are related to low malignancy of type I endometrial cancer, suggesting that these molecules could have dual oncogenic and cancer-suppressive effects in endometrial cancer cells. Another important protein, often evaluated to assess an aetiology-specific therapy in type 1 EC patients, is ERα [62]. Its amplification was regarded as a prevalent event in endometrial cancer, however, a decreased ERα level has been detected in high-stage and poorly differentiated cancers [63,64,65]. ERα seems to be suppressed by miR-206, leading to an anti-proliferation effect and decreased invasion capacity [66]. IGF1R, by binding its IGF1 and IGF2 ligands, is able to activate the PI3K pathway, stimulating cell proliferation and inhibiting apoptosis. Therefore, if it is over-expressed, as in most malignant tissues, it plays an anti-apoptotic role, promoting cancer cell survival and tumour metastasis [67,68]. Recent studies demonstrated that IGF1R was highly expressed in EC tissues and it was inversely correlated with miR-381 levels [69].

Epigenetic mechanisms seem to be involved in EC: silencing of DNA mismatch repair genes by DNA hypermethylation has been demonstrated [70]. Moreover, the study of methylation profiles in endometrial tumorigenesis showed that, among different tumour suppressor genes, the number of methylated promoters increased in the progression of cancer [71]. For example, miR-129-2 seems to be related to microsatellite instability and mutL homolog 1 (MLH1) methylation, which is frequently observed in EC cells, suggesting an important role in early stages of carcinogenesis [72,73]. Usually, miR-203 suppresses tumour proliferation, invasion and metastasis, through the inactivation of ABL proto-oncogene 1 (ABL1) and BCR-ABL1 oncogenes. In hematopoietic tumours, it has been found silenced by the hypermethylation of its promoter [74]. Huang and collaborators found its hypermethylation in EC cells lines and they observed that it was associated with microsatellite instability and MLH1 methylation in primary endometrioid EC [75]. Mir-152 is a tumour suppressor, which inhibits tumour cell growth both in vitro and in vivo by repressing Cell Division Cycle 25B (CDC25B), an important cell cycle regulator [76]. It is able to target E2F Transcription Factor 3 (E2F3), DNA methyltransferase 1 (DNMT1), met proto-oncogene (MET) and Rapamycin-insensitive companion of mTOR (Rictor) and in EC cells it seems to be down-regulated through CpG hypermethylation, promoting cancer development and progression [77].

Several miRNAs that regulate EMT have been found deregulated in EC. EMT, important in implantation, is also a key process in oncogenesis and tumour metastasis; it has been mainly related to the expression of three markers: epithelial E-cadherin, mesenchymal vimentin and N-cadherin [78]. MiR-93 has been described as a tumour suppressor in ovarian cancer and more recently as an oncomiR in EC cells. MiR-93 up-regulation leads to a downregulated E-cadherin and increased N-cadherin expression [79]. In addition, this miRNA seems to be able to target FOXA1, a negative regulator of EMT, down-regulated in EC cells [79,80]. Another miRNA involved in EMT processes is miR-30c, which targets Metastasis-associated gene-1 (MTA1) inhibiting cell proliferation, metastasis and invasion in EC cells [81]. It is down-regulated both in type 1 and type 2 EC cells and its expression seems to be related to oestrogen concentration [82]. MiR-106b has been related to EMT, too: it has been associated with Twist family bHLH transcription factor 1 (TWIST1) targeting [83]. TWIST1 contributes to the EMT phenotype in EC cells improving cell invasion and it is more expressed in EC cells than in normal ones [84]. These findings are consistent with miR-106b downregulation in EC cells showing EMT phenotypes; profiling its expression levels might be helpful for predicting the risk of metastasis, especially in patients with type II EC [83].

### 3.3. Circulating miRNA and Endometrial Cancer

To discriminate the different subtypes and predict prognosis by using circulating miRNAs as non-invasive biomarkers of EC, several investigations have investigated their expression profiles in serum or plasma. High serum levels of miR-155 have been found associated with cancer stage and lymph node metastasis [85]. Another study demonstrated that the association of miR-99a and miR-199b, up-regulated in plasma of EEC patients, better discriminate EEC patients [86]. A recent study demonstrated that down-regulated miR-29b expression in plasma correlates with poor EC prognosis and is helpful to evaluate the EC prognosis [87]. A genome-wide study on miRNA expression profiles from plasma of EEC and subsequent validation by quantitative reverse-transcriptase polymerase chain reaction demonstrated the significant up-regulation of 5 miRNAs (miR-15b, miR-27a, miR-223, miR-3145 and miR-4638). Moreover, the authors found that miR-27a and CA125 together have a considerable clinical value in diagnosing EEC [88].

In summary, a lot of miRNAs, cellular and circulating, have been found dysregulated in endometrial cancer and a comprehensive list and the relative references are reported in Table 2 [89,90,91,92,93,94,95,96,97,98,99,100,101,102,103,104,105,106,107,108,109,110,111,112,113,114].

### 3.4. lncRNAs in Endometrial Cancer

To date, there are no studies about lncRNA profiles during physiological endometrial changes but their altered expression in EC as well as in endometriosis has been demonstrated [115,116,117]. In fact, different lncRNAs seem to be differentially expressed in EC and some of them could have a prognostic value. Ovarian adenocarcinoma amplified lncRNA (OVAAL) is overexpressed in many ovarian serum carcinoma, as well as in type 1 EC but less frequently in type 2 EC [46,118]. Its over expression is related to overexpression of p53-regulated genes [118]. Metastasis-associated lung adenocarcinoma transcript 1 (MALAT1) is an 8000 nt lncRNA, upregulated in many different human tumours. It has been positively associated with hyperplasia and negatively with metastasis, thus it can be used as a predictive biomarker. Its expression is regulated by the Wnt/β-catenin signalling pathway, frequently abnormally enhanced in endometrioid type EC. Particularly, MALAT1 has been identified as a functional downstream target of Protocadherin 10 (PCDH10), a tumour suppressor protein, down-regulated in EEC [119]. Growth arrest-specific 5 (*GAS5*) is a tumour suppressor gene, implicated and aberrantly expressed in multiple cancers [120,121,122,123,124]. Recent evidences show that this lncRNA is down-regulated in endometrial cancer cells, being able to induce their apoptosis by inducing PTEN expression through inhibiting miR-103, which usually stimulates cell growth and invasion in endometrial carcinoma [125]. HOX transcript antisense intergenic RNA (HOTAIR) is one of the most studied lncRNAs, because of its involvement in genome modification: in fact, it can repress gene expression through the activation of chromatin modifiers [126]. Its overexpression is associated with increasing oestrogen levels and correlated with poor cancer prognosis. HOTAR has been found upregulated in EC compared to normal endometrium samples. HOTAIR overexpression is also associated with increased metastatic spread and a reduced overall-survival rate in EC [127]. Using a mouse xenograft EC model, treated with HOTAIR siRNA lentivirus, a significant tumorigenesis rate and tumour size reduction occurred both in vitro and in vivo [128]. This shows that HOTAIR may be a prognostic molecular marker for EC, even if further studies are required to demonstrate its involvement in endometrial cancer progression and metastasis [127] H19, a paternally imprinted lncRNA, is located on chromosome 11 and lies within 200 kbp downstream of the *IFG2* gene [129]. H19 is also known as “oncofetal non-coding RNA” because it is primarily expressed during foetal development, so it is absent or poorly expressed in most normal adult tissues. Its re-expression has been detected in various cancers, including ovarian cancer [130,131,132,133]. H19 expression levels increase throughout endometrial epithelium tumorigenesis: they are low in normal epithelium, higher in hyperplastic endometrium, very high in EC and even higher in dedifferentiated tumour tissues [115]. Urothelial Cancer-Associated 1 (UCA1) is one of the lncRNAs mostly associated with tumour progression, metastasis and chemo-resistance in several cancer types [134,135,136,137,138]. Lu et al. discovered that UCA1 levels were significantly higher in EC cells than in normal endometrial samples and in metastatic EC they noticed a further level increase if compared to primary tumour, suggesting its involvement in tumour cell migration [139]. The circRNAs, another class of ncRNAs, has been shown to be enriched and stable in exosomes. They act as natural miRNA sponges to decrease miRNA levels, reducing their regulatory effect on mRNAs [140,141]. In a recent study, Xu et al. found that the number of exosomes isolated from serum of EC patients was higher than those of normal samples. In addition, the expression profile of circRNAs in serum of EC patient was dysregulated [142]. Another analysis found several circRNAs differentially expressed in EC cells when compared to normal endometrium [143]. In Table 3, we present a list of significant lncRNAs deregulated in EC and the relative references [144,145,146,147,148,149,150,151,152,153,154,155,156].

## 4. ncRNAs and Endometriosis

### 4.1. Endometriosis

Endometriosis is a disabling disorder characterized by the presence of endometrial tissues outside of the uterine cavity. The most common symptoms are non-menstrual pelvic pain, dyspareunia and infertility [157]. The exact causes are unknown, even if the most shared theory is retrograde menstruation, that is endometrial cells through menstrual blood move in on pelvic cavity. These cells present different characteristics, as the ability of adhesion, aggression, neo-angiogenesis and inhibition of apoptosis, which make endometriosis similar to cancer [158,159]. 

Although decades of research about the pathogenesis of endometriosis have shown the role of hormonal and non-hormonal mechanisms related to disease development, the therapeutic approach and the methods for early diagnosis are still lacking. Today, the best method for the diagnosis of endometriosis remains laparoscopic surgery but the finding new biomarkers for a minimally invasive diagnosis represent an important scientific challenge [160]. 

### 4.2. miRNAs and Endometriosis

Different papers have been published about miRNAs potentially involved in endometriosis but unfortunately, many times the concordance among the results is small. This could be due to the heterogeneity of cell types used in the experiments, as illustrated in a recent review [161]. Another possible explanation is that various technologies (deep-sequencing, microarray, Real-Time PCR) have been used by different researchers.

The first studies on miRNAs and endometriosis demonstrated that different miRNAs were differentially expressed in endometrial tissues with endometriotic lesions with respect to endometrial tissues of the same woman without endometriotic lesions [162,163]. Burney et collaborators were among the first authors to publish the difference in expression profiles of miRNAs between eutopic endometrial tissues of woman with and without endometriosis [164]. They found that four miRNAs (miR-34c-5p, miR-9, miR-9*, miR-34b*) were down-regulated in women with endometriosis compared to controls. In another paper, the authors showed that in patients with ovarian endometriosis, miR-483-5p, targeting IGF2 and miR-629-3p were down-regulated in the eutopic endometrium. These authors suggest that the dysregulation of these miRNAs and their target genes could contribute to the overgrowth of endometrial tissue outside the uterus [165]. Further studies have been carried out in the last few years on miRNAs and endometriosis. In 2017, Min Kyoung Kim and collaborators demonstrated that the expression of miR-27b-3p, upregulated in human endometrial stromal cells from patients with endometriosis, is reduced after the treatment with Rg3-enhanced red ginseng extract (Rg3E). The authors showed the same effects in vivo in a mouse model [166]. In 2018, by RNA sequencing, it was demonstrated that 107 miRNAs and 6112 mRNAs were differentially expressed in ectopic endometrium. The authors built regulatory networks among Transcription Factors, miRNAs and mRNA targets identifying the most important points of altered regulation in ectopic tissue. Among the miRNAs, they found that some members of miR-449 and miR-34b/c cluster, miR-200 family, miR-106a-363 cluster were dysregulated [167] (Table 4). Members of miR-200 family are also involved in cell migration and EMT which supposedly occurs in the pathogenesis of endometriosis [161]. The downregulation of members of miR-200 family in endometrial tissues with endometriotic lesion results in increased expression of ZEB1/ZEB2 which are transcriptional repressors of E-cadherin. E-cadherin, in the normal endometrial tissue, is required for maintaining the epithelial nature of cells and their adhesion. Its down-regulation leads to epithelial cells to acquire mesenchymal characteristics [161].

Neoangiogenesis regulators such as Vascular Endothelial Growth Factor A (VEGFA) and Thrombospondin-1 (THBS1) have been involved in the pathology of endometriosis [168]. Two different groups of researchers reported that miR-17-5p and miR-20a were down-regulated in the ovarian endometrium compared to eutopic endometrium [165,169] Braza-Boïls and collaborators, in 2013, reported that the miR-17-92 cluster increases tumour neovascularization by decreasing THBS1 expression [170]. MiR-15a-5p, a negative regulator of VEGFA, has been found down-regulated in endometrium samples of woman with endometriosis and VEGFA was found up-regulated in the same endometrial tissues. Moreover, the transfection of endometriosis stromal cells with miR-15a-5p mimics led to reduction in the expression of VEGFA and migration abilities of the endometrial stromal cells, suggesting an important role of this miRNA in the pathogenesis of endometriosis [171].

It has been demonstrated that endometriotic cells in vitro are able to secrete miRNA EV cargo, in culture medium and the role of extracellular miRNAs in the pathogenesis of endometriosis has also been studied. Harp and collaborators observed that miR-21, pro-angiogenic miRNA, is up-regulated in exosomes from endometriosis samples compared with exosomes from controls. The authors suggested that exosomes play autocrine/paracrine roles in the development of endometriosis, potentially by modulating angiogenesis [172].

### 4.3. Circulating miRNAs as Biomarkers of Endometriosis

Due to the anatomical location of this disease, several closely related biological fluids have been proposed as source for non-invasive biomarkers of endometriosis, for example: urine, plasma/serum and menstrual blood. One potential use of circulating miRNAs as non-invasive biomarkers for endometriosis is an ongoing area of research and its diagnostic and therapeutic implications have been extensively reviewed [164,173,174,175]. In 2013, Wang and collaborators performed a circulating miRNA array profiling in serum and they found different deregulated miRNAs [176] (Table 4). Moreover, a microarray analysis revealed that miR-17-5p, miR-20a and miR-22 were down-regulated in plasma from patients with endometriosis [177]. It is known that prognostic value increases when two or more biomarkers are evaluated at the same time, in fact, two studies performed in serum and plasma demonstrated that let-7b, let-7d and let-7f altered levels, as well as miR-200a-3p, miR-200b-3p and miR-141-3p down-regulation are able to discriminate patients with endometriosis from controls [160,178] (Table 4).

### 4.4. lncRNAs and Endometriosis

In a paper published in 2015, Wang and collaborators compared the expression of several lncRNAs and mRNAs between eutopic and normal endometrium by microarray analysis and validated the obtained data using RT-qPCR. The authors found several lncRNAs and mRNAs differentially expressed between eutopic and normal endometrium. Specifically, among the lncRNAs, 488 were up-regulated and 789 down-regulated and among the mRNAs, 578 were over expressed and 638 down-regulated [117]. The most significantly up-regulated lncRNA is AC068282.3 (fold change 31.3) while RP11-403H13.1 is the most significantly down-regulated one (fold change 44.3). The differentially expressed lncRNAs were involved in cell cycle regulation and immune response [117]. As previously described, H19 is a lncRNA, which is expressed from the imprinted locus that also contains the reciprocally imprinted IGF2 gene. It has been shown that H19, reducing the bioavailability of miRNA let-7, by acting as a molecular sponge, was significantly down-regulated in the eutopic endometrium of women with endometriosis. Its down regulation, increasing let-7 activity, inhibited IGF1R expression. Accordingly, the proliferation of endometrial stromal cells was reduced. This paper represents the first example of lncRNA involvement in the pathogenesis of endometriosis and its association with infertility [179].

Another lncRNA that is important in endometriosis is MALAT1. This is one of the most known lncRNAs, it is evolutionary well-conserved and is overexpressed in many cancers [180,181,182,183]. Recently, a paper demonstrated that the expression of MALAT1 is significantly increased during endometriosis. Moreover, the authors showed that miR-200c, which is regulated by MALAT1, was significantly down-regulated in endometrial samples from woman with endometriosis and they were significantly negatively correlated. The functional assay showed that overexpression of miR-200c inhibited the proliferation of Human Endometrial Stromal Cells (HESCs) while the inhibition of miR-200c promoted cellular proliferation. Similar results were also obtained from the migration assay in which MALAT1 knockdown inhibits the proliferation and migration of HESCs [184]. Finally, the presence of deregulated lncRNAs in serum as potential non-invasive biomarkers of endometriosis has been investigated. In 2016, it has been reported that a set of lncRNA in serum can discriminate severe versus mild stages of endometriosis and other associated clinical features. Moreover, the combination of five lncRNAs (NR_038395, NR_038452, ENST00000482343, ENST00000544649, ENST00000393610) is able to discriminate patients with and without endometriosis [116]. In Table 5, we show the list of significant lncRNAs deregulated in endometriosis and the relative references.

## 5. ncRNAs and Chronic Endometritis

### 5.1. Chronic Endometritis

Chronic Endometritis (CE) is a chronic inflammation of the endometrium that is difficult to diagnose, caused by microbial infection sustained by common bacteria such as Escherichia coli, Enterococcus faecalis and Streptococcus agalactiae [185,186]. It has vague symptoms, such as abnormal uterine bleeding, pelvic pain and leukorrhea and, at the moment, hysteroscopy represents the most reliable diagnostic method [187,188]. In spite of the benign prognosis, this pathology can impair endometrial receptivity, in spontaneous and in IVF cycles [189,190]. Moreover, there are evidences showing that CE could be a cause of natural preterm labour, premature birth and recurrent miscarriages [188,191]. To date, the molecular mechanisms with which CE can cause infertility still remain unknown.

In a paper published by our group in 2013, we demonstrated the altered endometrial expression of genes, involved in inflammatory response, proliferation and apoptosis in CE. These findings are in agreement with literature data, showing that the endometrium of CE women results an unusual local microenvironment, due to an altered secretion of paracrine factors [192,193,194]. Accordingly, endometrial receptivity may be impaired as well as proliferative processes may be increased [195,196].

### 5.2. miRNAs and Chronic Endometritis

At the moment, very few data are present in the literature about the alteration of molecular pathways associated with CE. In spite of CE is considered a mild pathology, it is related to infertility and it has been suggested the association between CE and endometriosis and between CE and the endometrial micro-polyps [193,195]. Therefore, to investigate the alteration of molecular mechanisms caused by a chronic infection could be interesting.

**Figure 3:**
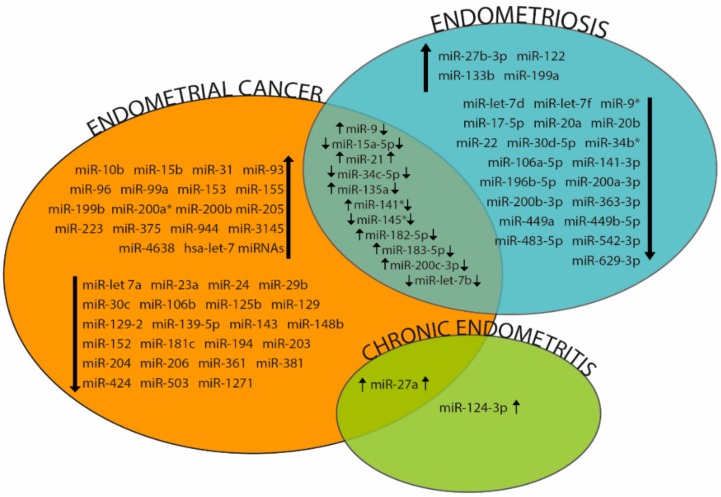
Representative miRNAs involved in endometrial pathologies.

We showed, for the first time, an up-regulation of miR-27a-3p and miR-124-3p in the endometrium from women affected by CE and demonstrated their ability to discriminate CE women when the 2 miRNAs were analysed together in the serum [197]. Previously, miR-27a has been found highly expressed in cows with subclinical endometritis [198]. Finally, we found that miR-27a up-regulation is significantly related to IGF1 down-regulation in the same endometrial samples [197]. Proper IGF1 levels are required for successful embryonic and placental development; consequently, the down-regulation of protein could be associated with endometrial quality and with infertility observed in women with CE [199]. Interestingly, miR-27a and miR-27b, two isoforms of the miR-27 family, seem to be involved in different endometrial pathologies. Their up-regulation in endometrium and serum has been described in EC, in endometriosis and in CE [56,166,197] (Figure 3). It would be attractive to demonstrate, by functional experiments, its ability to regulate IGF1 and other messengers, in order to understand its role in endometrial physiopathology and evaluate its prognostic potential and the possibility to plan specific therapy based on RNA interference.

**Figure 4:**
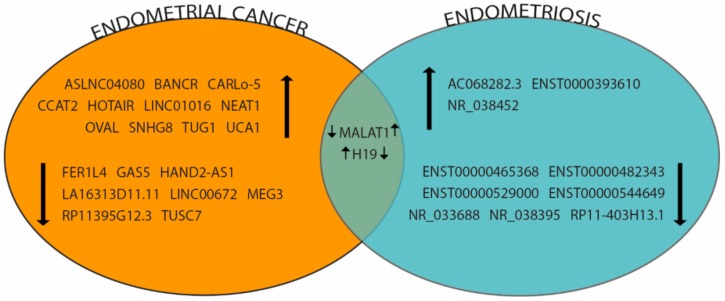
Representative lncRNAs involved in endometrial pathologies.

Literature analysis revealed that there are no data about lncRNAs in CE (Figure 4). The overlapping among lncRNAs involved in EC and Endometriosis showed that MALAT1 and H19 are shared across the two pathologies, even if, they show a different trend of expression (Figure 4).

Venn diagram summarizing deregulated miRNAs identified in Endometrial Cancer, Endometriosis and Chronic Endometritis. Intersection areas show molecules differentially expressed in more than one pathology. Up and down-regulated miRNA expression is indicated by black arrows.

Venn diagram summarizing deregulated lncRNAs identified in Endometrial Cancer and Endometriosis. Intersection areas show molecules differentially expressed in more than one pathology. Up and down-regulated lncRNA expression is indicated by black arrows.

## 6. Conclusions

The discovery of ncRNAs in the regulation of gene expression inside the pathway from genotype (DNA) to phenotype (Proteins) has deeply transformed contemporary biomedicine. The countless studies on pathogenesis of tumours and complex phenotypes revealed the involvement of ncRNAs in different human diseases and postulated their role as molecular biomarkers in diagnosis, prognosis and also as possible therapeutic targets. In this review, we reported that several ncRNAs are involved in endometrial physiology and that their altered expression can be related to different endometrial disorders. High-throughput techniques are able to give a general overview, discovering many molecules involved in a specific biological pathway. Nevertheless, the complexity of ncRNA networks makes difficult to understand if deregulation of specific molecules represents the etiological cause of a disease or, alternatively, a secondary effect, depending on the perturbation of physiological pathways. Even if, EC, endometriosis and CE are dissimilar endometrial disorders, an association among them could be supposed. Comparing literature data, we detected ncRNAs found dysregulated in the three pathologies (Figure 3 and Figure 4). For some of them, there is no expression concordance between EC and Endometriosis, probably due to the different regulated targets but interestingly 4 miRNAs show the same expression trends and the up-regulation of miR-27 family seems to be involved also in CE. We believe that in order to understand and resolve particular biomedical problems, it would be appropriate to focus on a limited number of molecules to perform functional analyses, in vitro and in vivo on animals.

## Figures and Tables

**Table 1:** miRNAs involved in embryo receptivity.

miRNAs	Species	Sample	Reference
mmu-let-7a	mouse	endometrium	[23]
mmu-let-7b	mouse	endometrium	[23]
hsa-let-7e*	human	exosomes from endometrial cell lines	[34]
hsa-let-7f-2*	human	exosomes from endometrial cell lines	[34]
hsa-miR-30b	human	endometrium	[24]
hsa-miR-30d	human	endometrium/endometrial fluid	[24,32]
hsa-miR-122	human	exosomes from endometrial cell lines	[34]
hsa-miR-124	human	exosomes from endometrial cell lines	[34]
hsa-miR-125b	human	endometrial cell lines	[30]
mmu-miR-126-3p	mouse	endometrium	[29]
hsa-miR-129*	human	exosomes from endometrial cell lines	[34]
hsa-miR-142-3p	human	exosomes from endometrial cell lines	[34]
hsa-miR-145	human	endometrium	[26]
mmu-miR-199a	mouse	endometrium	[25]
hsa-miR-222*	human	exosomes from endometrial cell lines	[34]
hsa-miR-376c	human	exosomes from endometrial cell lines	[34]
hsa-miR-409-3p	human	exosomes from endometrial cell lines	[34]
mmu-miR-429	mouse	endometrium	[28]
hsa-miR-432	human	exosomes from endometrial cell lines	[34]
hsa-miR-451	human	exosomes from endometrial cell lines	[34]
hsa-miR-494	human	endometrium	[24]
hsa-miR-520h	human	exosomes from endometrial cell lines	[34]
hsa-miR-1248	human	exosomes from endometrial cell lines	[34]

**Table 2:** miRNAs differentially expressed in endometrial cancer (EC) patients.

miRNAs	Expression	Sample	Reference
Has-let-7 miRNAs	up	EEC	[61]
miR-let-7a	down	EC	[89]
miR-let-7b	down	EC	[90]
miR-9	up	EEC	[56]
miR-10b	up	EC	[91]
miR-15a-5p	down	EC	[92]
miR-15b	up	Plasma in EEC patients	[88]
miR-21	up	EEC	[53]
miR-23a	down	EEC	[93]
miR-24	down	EC	[94]
miR-27a	up	EEC, Plasma in EEC patients	[56,88]
miR-29b	down	EC, Plasma	[87,95]
miR-30c	down	EC	[81,82]
miR-31	up	EC	[96]
miR-34c	down	EC	[97]
miR-96	up	EEC	[56]
miR-99a	up	Plasma in EEC	[86]
miR-106b	down	EEC	[83]
miR-125b	down	EEC	[98]
miR-129	down	EC	[99]
miR-129-2	down	EC	[72]
miR-135a	up	EC	[100]
miR-139-5p	down	EC	[101]
miR-141	up	EEC	[102]
miR-143	down	EC	[103]
miR-145	down	EEC	[104]
miR-148b	down	EC	[105]
miR-152	down	EC	[76]
miR-153	up	EC	[56]
miR-155	Up	Serum	[85]
miR-181c	down	EEC	[106]
miR-182-5p	up	EEC	[56,102]
miR-183-5p	up	EC	[108]
miR-194	down	EC	[109]
miR-199b	up	Plasma in EEC patients	[86]
miR-200a*	up	EC	[102,108]
miR-200b	up	EEC	[102]
miR-200c-3p	up	EC	[110]
miR-203	down	EC	[74]
miR-204	down	EC	[57]
miR-205	up	EEC	[50,51,102]
miR-206	down	EC	[66]
miR-223	up	Plasma in EEC	[88]
miR-361	down	EC	[90]
miR-375	up	EC	[100]
miR-381	down	EC	[69]
miR-424	down	EC	[111]
miR-503	down	EEC	[112]
miR-944	up	EC	[113]
miR-1271	down	EC	[114]
miR-3145	up	Plasma in EEC patients	[88]
miR-4638	up	Plasma in EEC patients	[88]

**Table 3:** lncRNAs differentially expressed in EC patients.

lncRNAs	Expression	EC Type	Reference
ASLNC04080	up	EC	[144]
BANCR	up	EEC	[145]
CARLo-5	up	EC	[146]
CCAT2	up	EC	[147]
FER1L4	down	EC	[148]
*GAS5*	down	EC	[125]
H19	up	EC	[115]
HAND2-AS1	down	EEC	[149]
HOTAIR	up	EC	[127]
LA16313D11.11	down	EC	[150]
LINC00672	down	EC	[151]
LINC01016	up	EC	[152]
MALAT1	down	EEC	[119]
MEG3	down	EC	[153]
NEAT1	up	EEC	[154]
OVAL	up	EEC	[46,118]
RP11395G12.3	down	EC	[150]
SNHG8	up	EC	[155]
TUSC7	down	EC	[156]
UCA1	up	EC	[139]

**Table 4:** miRNAs differentially expressed in Endometriosis patients.

miRNAs	Expression	Sample	Reference
let-7b	down	serum	[160]
let-7d	down	serum	[160]
let-7f	down	serum	[160]
miR-9	down	endometrium	[164]
miR-9*	down	endometrium\serum	[164,176]
miR-15a-5p	down	endometrium	[171]
miR-17-5p	down	ovarian endometrium\plasma	[165,169,177]
miR-20a	down	ovarian endometrium\plasma	[165,169,177]
miR-20b-5p	down	endometrium	[167]
miR-21	up	endometrial exosomes	[172]
miR-22	down	plasma	[175]
miR-27b-3p	up	endometrium	[166]
miR-30d-5p	down	endometrium	[167]
miR-34b*	down	endometrium	[164]
miR-34c-5p	down	endometrium	[164]
miR-106a-5p	down	endometrium	[167]
miR-122	up	serum	[176]
miR-133b	up	endometrium	[167]
miR-135a	down	serum	[160]
miR-141*	down	serum	[176]
miR-141-3p	down	endometrium\plasma	[167,176]
miR-145*	down	serum	[176]
miR-182-5p	down	endometrium	[167]
miR-183-5p	down	endometrium	[167]
miR-196b-5p	down	endometrium	[167]
miR-199a	up	serum	[176]
miR-200a-3p	down	endometrium\plasma	[161,167,178]
miR-200b-3p	down	endometrium\plasma	[161,167,178]
miR-200c-3p	down	endometrium	[161,167]
miR-363-3p	down	endometrium	[167]
miR-449a	down	endometrium	[167]
miR-449b-5p	down	endometrium	[167]
miR-483-5p	down	endometrium	[165]
miR-542-3p	down	serum	[176]
miR-629-3p	down	endometrium	[165]

**Table 5:** lncRNAs differentially expressed in Endometriosis patients.

lncRNAs	Expression	Sample	Reference
AC068282.3	up	endometrium	[117]
ENST00000393610	up	serum	[116]
ENST00000465368	down	serum	[116]
ENST00000482343	down	serum	[116]
ENST00000529000	down	serum	[116]
ENST00000544649	down	serum	[116]
H19	down	endometrium	[179]
MALAT1	up	endometrium	[184]
NR_033688	down	serum	[116]
NR_038395	down	serum	[116]
NR_038452	up	serum	[116]
RP11-403H13.1	down	endometrium	[117]
